# Hybrid Management of an Aortobronchial Fistula after Patch Aortoplasty for Aortic Coarctation in a Patient with SARS-CoV-2 Pneumonia: Case Report and Review of the Literature

**DOI:** 10.3390/medicina58101385

**Published:** 2022-10-02

**Authors:** Grigore Tinica, Andrei Tarus, Alberto Bacusca, Raluca Ozana Chistol, Alexandra Cristina Rusu, Mihaela Tomaziu Todosia, Cristina Furnica

**Affiliations:** 1 Faculty of Medicine, “Grigore T. Popa” University of Medicine and Pharmacy, 700115 Iasi, Romania; 2 Cardiovascular Diseases Institute, 700503 Iasi, Romania; 3 Department of Ophthalmology, Targu Mures County Hospital, 540072 Targu Mures, Romania; 4 Doctoral School of Medicine and Pharmacy, University of Medicine, Pharmacy, Science and Technology “George Emil Palade”, 540139 Targu Mures, Romania; 5 Department of Institutional Development, “Grigore T. Popa” University of Medicine and Pharmacy, 700115 Iasi, Romania; 6 Research Center in Management, Alexandru Ioan Cuza University, 11 Carol I Blvd., 700506 Iasi, Romania; 7 Institute of Forensic Medicine, 700455 Iasi, Romania

**Keywords:** hemoptysis, aortobronchial fistula, aortic coarctation, patch repair, pseudoaneurysm, thoracic endovascular aneurysm repair, hemi-aortic arch debranching, SARS-CoV-2 pneumonia

## Abstract

Aortobronchial fistula is a rare cause of repeated hemoptysis and a potentially fatal condition if left untreated. We present the case of a 40-year-old man with repeated hemoptysis, excessive cough, and epistaxis ongoing for several days after SARS-CoV-2 pneumonia diagnosis. The patient had a history of patch aortoplasty for aortic coarctation and aortic valve replacement with a mechanical valve for aortic insufficiency due to bicuspid aortic valve at the age of 24. Computed tomography scan performed at presentation revealed a severely dilated ascending aorta, a thoracic aorta pseudoaneurysm at the site of the former coarctation, an aortobronchial fistula suggested by the thickened left lower lobe apical segmental bronchus in contact with the pseudoaneurysm and signs of alveolar hemorrhage in the respective segment. The patient was treated with thoracic endovascular aneurysm repair (TEVAR) after prior hemi-aortic arch debranching and transposition of the left common carotid artery and subclavian artery through a closed-chest surgical approach. Our case report together with a systematic review of the literature highlight the importance of both considering an aortobronchial fistula in the differential diagnosis of hemoptysis in patients with prior history of thoracic aorta surgical intervention, regardless of associated pathology, and of taking into account endovascular and hybrid techniques as an alternative to open surgical repair, which carries a high risk of morbidity and mortality.

## 1. Introduction

Aortobronchial fistulas (ABF) are rare, abnormal, and potentially life-threatening communications between the descending thoracic aorta and the tracheobronchial tree. They are known to occur secondary to traumatic thoracic aorta injuries [1], penetrating aortic ulcers [2], mycotic aneurysms [3], or as distant complications to previous aortic surgery [4,5]. Exceptionally, cases secondary to iatrogenic aortic injury during percutaneous coronary intervention have also been reported [6].

Given the wide range of aortic pathologies that could underlie such a condition, the diagnosis should be considered for any patient with hemoptysis and prior history of aortic surgery. No case has been reported in association with SARS-CoV-2 pneumonia, to date.

Traditional management of ABF consists of open surgical repair of the aorta and, if necessary, reconstruction of the trachea or involved bronchus, a complex procedure associated with increased mortality rates of up to 41% [7,8].

A less invasive approach emerged in 1994 when Dake et al. reported the first case series (13 patients) of endovascular stent grafting of descending thoracic aorta aneurysms [9]. Since then, the procedure has become a mainstay therapy in the treatment of various aortic diseases.

Stent-grafts usually require bilateral landing zones of at least 20 mm. In the case of lesions close to the ostia of the supra-aortic vessels, a landing zone needs to be created in the aortic arch through arterial debranching and transposition (varying from one, two, or all three arteries).

We present the successful repair of an ABF in a patient diagnosed with SARS-CoV-2 pneumonia and history of patch aortoplasty for aortic coarctation using a self-expandable stent-graft, following closed-chest surgical debranching of the left common carotid artery and left subclavian artery together with a systematic review of the literature concerning the diagnosis and management of ABF secondary to aortic coarctation repair.

## 2. Case Report

A 40-year-old male patient presented to the emergency department of a county hospital with fatigability, dyspnea, cough, hemoptysis, and epistaxis ongoing for several days. The symptoms began three weeks after being diagnosed with SARS-CoV-2 pneumonia with moderate lung involvement on chest X-ray.

A past repair (at the age of 24) of severe aortic coarctation and bicuspid aortic valve was reported by the patient who also provided a discharge letter from another cardiac surgery department mentioning Dacron patch aortoplasty for a severe aortic coarctation and replacement of an insufficient bicuspid aortic valve with a 25 mm Medtronic-Hall mechanical valve (Medtronic, Minneapolis, MN, USA). Since the surgical intervention, he was diagnosed and treated for essential arterial hypertension and NYHA (New York Heart Association) class III heart failure and also received antiaggregant and anticoagulant drugs to prevent valve thrombosis.

Given the recent history of SARS-CoV-2 pneumonia with moderate lung involvement, the suspicion of pulmonary thromboembolism was raised, requiring pulmonary computed tomography angiography (CTA) for exclusion. The radiological findings revealed interstitial pneumonia compatible with SARS-CoV-2 etiology, a dilated ascending aorta, and an aortic pseudoaneurysm (PSA) after the origin of the left subclavian artery with signs of active bleeding and no pulmonary embolism. The patient was then referred to our institution for further diagnosis and treatment.

**Figure 1:**
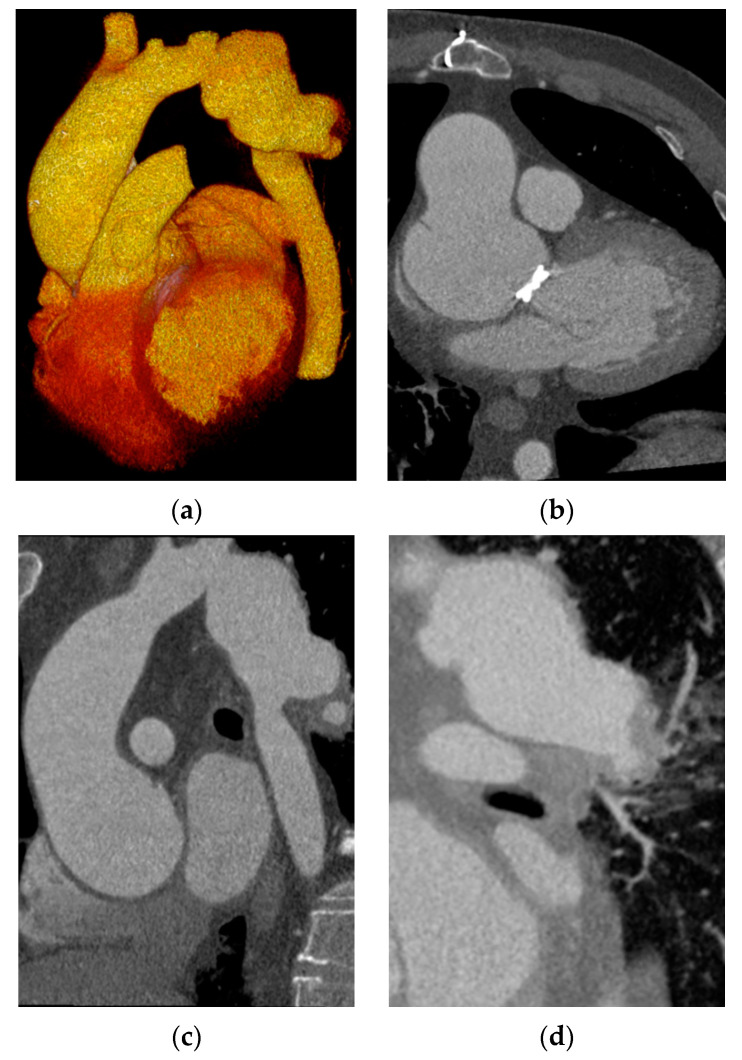
Cardiac CT angiography: (**a**) 3D VRT (volume rendering technique) reconstruction of the aorta showing the PSA; (**b**) closed mechanical valve and dilated ascending aorta with chronic limited dissection; (**c**) pseudoaneurysm of the descending thoracic aorta immediately after the ostium of the left subclavian artery; (**d**) thickened left lower lobe apical segmental bronchus in contact with the PSA and adjacent alveolar hemorrhage.

Because of the low quality of the first CTA, an EKG-gated cardiac computed tomography angiography (CCTA) was performed at admission in our institution and revealed a functional mechanical valve, severely dilated aortic root, and ascending aorta (63.3 mm) with a limited chronic dissection of the ascending aorta and an irregular PSA (80.6 × 40 × 38.4 mm) immediately after the origin of the left subclavian artery, the situs of the initial aortic coarctation, with no more active bleeding but in contact with a thickened left lower lobe apical segmental bronchus and signs of alveolar hemorrhage in the respective segment (Figure 1a–d).

As the patient was diagnosed with SARS-CoV-2 pneumonia, a redo surgery requiring a long duration of anesthesia was deemed too high risk. In his case, the heart team opted for a hybrid repair of the aortic arch and proximal descending thoracic aorta as the proximity of the PSA to the ostia of the left subclavian artery and left common carotid artery did not allow a correct deployment of an endovascular graft.

The hybrid repair was performed in a single intervention with surgical debranching and transposition of the left common carotid artery and left subclavian artery to the right common carotid artery as the first step. The left subclavian artery and left common carotid artery were accessed via a supraclavicular approach with transection of the anterior scalene muscle, and the right common carotid artery via a classical approach parallel to the anterior border of the sternocleidomastoid muscle. Both the left subclavian artery and left common carotid artery were ligated and transected. A single 8-mm Dacron graft was used for debranching, with a 1st end-to-side anastomosis to the right common carotid artery, a 2nd end-to-end anastomosis to the left subclavian artery, and a 3rd end-to-side anastomosis of the left common carotid artery to the graft. Through this technique, an adequate landing zone was prepared for the aortic graft.

**Figure 2:**
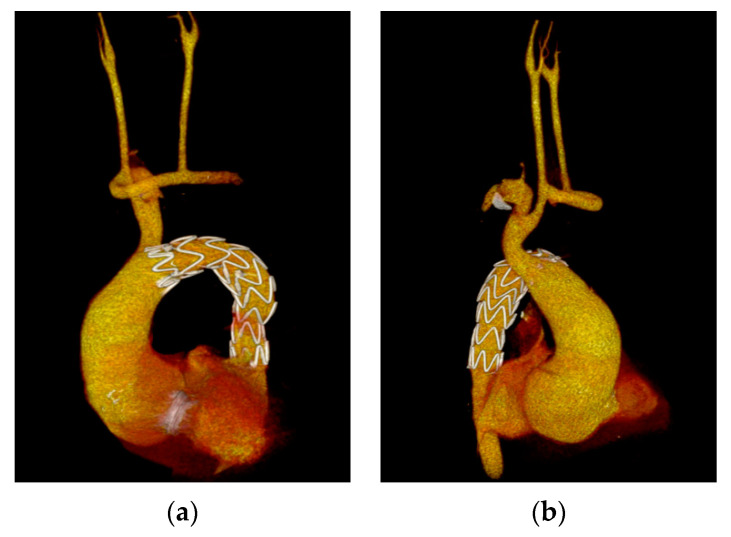
Cardiac CT angiography: (**a**) 3D VRT reconstruction showing successful exclusion of the PSA with no residual opacification; (**b**) 3D VRT reconstruction illustrating the transposed left common carotid and subclavian arteries using synthetic grafts.

The next step involved inserting a 28 × 28 × 157 mm Valiant™ Captivia Stent Graft with the Captivia™ Delivery System (Medtronic, Minneapolis, MN, USA) via the abdominal aorta (approached through laparotomy) under fluoroscopic control as both femoral arteries were hypoplastic because of the aortic coarctation. The landing zone was tangent to the ostium of the brachiocephalic trunk and the stent-graft covered the ostia of the left common carotid artery and left subclavian artery and successfully excluded the pseudoaneurysm with no endoleak as confirmed by the control CCTA performed before discharge (Figure 2a-b).

Considering recent SARS-CoV-2 pneumonia, a second intervention to replace the ascending aorta was planned after complete recovery. The patient was contacted 6 months after discharge and stated that hemoptysis did not reoccur but refused to present to the hospital as he developed a depressive disorder.

## 3. Discussions

An ABF is a rare and potentially fatal condition if left untreated [6,10], most being caused by descending thoracic aorta PSAs eroding the trachea or a bronchus, thus leading to hemoptysis as the main symptom. The condition is frequently misdiagnosed especially in patients with comorbid conditions (our patient with SARS-CoV-2 pneumonia was initially suspected of pulmonary thromboembolism) and approximately 30% of cases are only confirmed at autopsy [11].

Our patient’s age and medical history (surgical correction of an aortic coarctation) could suggest the diagnosis. Fistulas occur at variable time intervals (between less than 1 year [12] and 37 years [13]) after the initial intervention, and, generally on the left side due to the proximity and adherences between the descending thoracic aorta and the left lung/left bronchial tree.

More than 15 years ago, synthetic patch aortoplasty was widely used worldwide to treat aortic coarctation, but the high rate of long-term complications and PSAs (in up to 32% of cases) has led to a gradual abandonment of the technique [14,15].

In our patient, the poorly controlled arterial hypertension together with excessive cough led to continuous pressure against the weakened aortic wall with subsequent PSA and fistulisation into a bronchus. Luckily, the breach was small enough not to cause massive hemoptysis, as mortality rates associated to massive hemoptysis reach 71% if more than 600 mL of blood are lost in 4 h. The mechanism of death in such cases involves acute airway obstruction with hypoxemic respiratory failure, hypotension, or blood loss [16].

The present paper illustrates a complex ABF variant due to an aortic PSA. The ABF was discovered on a CTA examination performed to exclude pulmonary thromboembolism. An open surgical approach was deemed inadequate because of ongoing SARS-CoV-2 pneumonia, the heart team opting for a less invasive hybrid approach with closed-chest surgical hemi-aortic arch debranching to create an adequate landing zone for stent graft placement in thoracic endovascular aortic repair (TEVAR). The stent graft was inserted via the abdominal aorta as the femoral arteries were hypoplastic and successfully excluded the PSA and the ABF, thus obtaining complete hemorrhage control.

Stent-graft infection is a major concern in cases with ABF [17], patients receiving prophylactic wide spectrum antibiotic therapy before, during and after the intervention. Eren et al. also suggest soaking the stent-graft in an antibiotic solution prior to implantation if there is a high suspicion of bacterial contamination of the ABF or if the diagnosis is delayed [18].

A close follow-up including control CT angiography is indicated to exclude endoleak, stent migration, and aneurysmal progression after TEVAR [19].

### Literature Review

To comprehensively discuss the therapeutic management of ABF following a prior repair of aortic coarctation, the authors performed a systematic review of reported cases to date.

**Figure 3:**
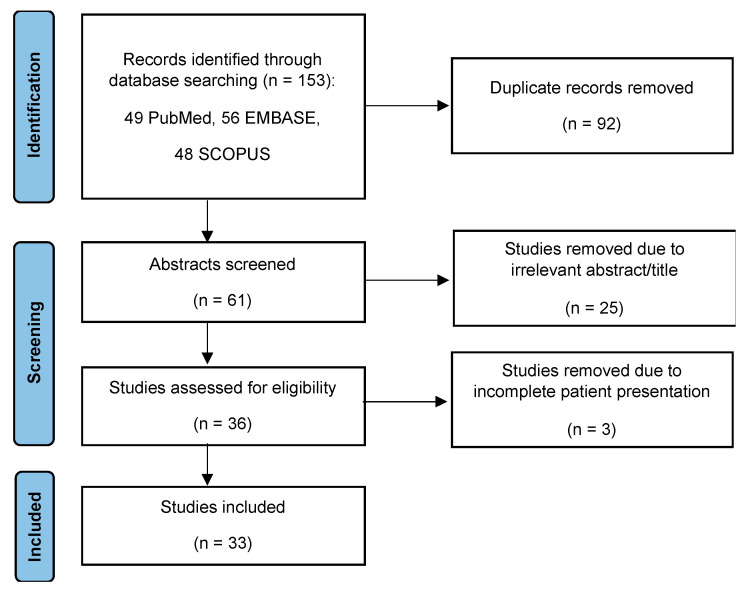
PRISMA flow chart of the selection process.

PubMed, EMBASE, and SCOPUS databases were searched on 8 August 2022 using the queries “aortic coarctation” and “aortobronchial fistula” returning the results displayed in Figure 3. We considered studies that met the following inclusion criteria: (1) Patients with ABF and a history of surgery for aortic coarctation; (2) articles identified as case reports, case series, letters to the editor, short communications, congress abstracts, correspondences containing case presentations or observational studies; and (3) articles written in English, French, Spanish, Portuguese or with an abstract in English, French, Spanish, Portuguese describing the case(s). We excluded (1) articles not presenting a case of ABF after prior surgery for aortic coarctation, (2) articles without an adequate abstract or full text available online, and (3) articles written in other languages with no abstract in English, French, Spanish, Portuguese describing the case.

A total of 153 articles were identified in the three databases. After removing 92 duplicates and 25 articles without an adequate abstract in English, French, Spanish, or Portuguese, two reviewers (CF, ROC) independently screened identified references (title and abstract) and additionally excluded three studies that did not meet inclusion criteria (Figure 3, Table 1) or proved to be of insufficient quality when evaluated using the tool developed by Murad et al. [20].

All but a single study [40] were in English. A total of 33 articles met inclusion criteria, comprising 37 cases.

Of the 37 patients who underwent coarctation repair and required reintervention for ABF as a complication of prior surgery, 24 (64.86%) were male and the mean (±SD) age across studies was 41.81 ± 15.76 years (5–78 years old). All patients presented hemoptysis of variable severity with cough, chest pain, dyspnea, and dysphonia as associated symptoms in a limited number of cases.

Like our case, most of the patients (25 cases—67.57%) had a single intervention for aortic coarctation correction while 12 patients (32.43%) had 1 or 2 reinterventions for complications of a previous surgery (Table 1).

The last (or single) intervention resulting in the ABF was performed 16.22 ± 9.73 years (16 years in case of our patient) before the complication occurred. In all cases, the ABF resulted from a PSA eroding a bronchus. The PSA occurred secondary to patch aortoplasty in 16 cases (43.24%), interposition of a synthetic graft in 7 cases (18.92%), extra-anatomic bypass in 8 cases (21.62%), or due to an unspecified intervention in 6 cases (16.22%). Marcheix et al. [37] and Manganas et al. [36] indicate a late PSA incidence post-patch aortoplasty ranging from 3 to 38%. This might suggest that Dacron patch aortoplasty should be used with caution, but no single technique is free of complication.

The PSA complicated with ABF was surgically treated in 20 cases (54.05%), by an endovascular approach in 14 cases (37.84%) and by a hybrid, endovascular, and surgical approach, in 2 cases (5.41%). A 5-years-old patient died of uncontrollable intrabronchial hemorrhage during surgery (Table 2).

The surgical treatment of the PSA complicated with ABF mainly consisted in interposition of a synthetic graft in 12 cases (32.43%) followed by extra-anatomic bypass in 3 cases (8.11%). Perioperative or long-term complications occurred in 5 of the 21 cases that benefited of a surgical treatment (23.81%) and in 5 of the 16 cases that benefited of an endovascular or hybrid approach (31.25%). There was no significant association between the therapeutic method and complications rate (chi-squared *p* = 0.613). On the other hand, complications were more severe with conventional surgery (MSOF, stroke, exitus) compared to the endovascular/hybrid approach.

The treatment of PSAs complicated with ABF gradually increased in complexity over time, from direct closure of the aorta [20], to interposition of synthetic grafts [22,24,27,29,30,31,38,44,45], extra-anatomic bypasses with or without resection of the native descending thoracic aorta [30,31,35], concomitant multiple endovascular procedures (TEVAR associated with coil embolization and occlusion with transcatheter devices) [23,41], and hybrid approaches combining TEVAR (±other endovascular procedures) with closed-chest debranching and surgical bypasses [12,13]. Our case is the 3rd hybrid approach reported and the first in a SARS-CoV-2 patient.

The short-, mid-, and long-term results over a period ranging from 30 days to 10 years revealed no difference between complications rate associated to surgical or endovascular approaches, but an increased severity of complications associated to open surgery.

The largest study concerning the outcome of surgical correction of aortic coarctation was performed by Knyshov et al. [47] who analyzed 891 patients and found 48 PSAs (5.4%) occurring at 1 to 24 years after aortic coarctation repair. Most of these cases (43 patients—89.6%) were treated by synthetic patch aortoplasty. Thirty patients (62.5%) were reoperated and 4 of these (13.8%) died after reoperation. All 18 patients (37.5%) that did not benefit from a reintervention died of ruptured PSA 7–15 years after coarctation repair.

The authors have chosen this case presentation to raise awareness on a rare but potentially fatal condition presenting with hemoptysis. All patients with a history of aortic surgery addressing for hemoptysis should benefit from a CTA to exclude an ABF, irrespective of known comorbidities as complications can occur as late as 37 years after the initial surgery [13]. In our patient with SARS-CoV-2 pneumonia, a pulmonary thromboembolism was initially suspected as part of the coagulopathy caused by SARS-CoV-2 [48]. SARS-CoV-2 pneumonia was not involved in the pathogenesis of the PSA but could have contributed to the development of the ABF by increased intrathoracic pressure caused by the excessive coughing. Endovascular and hybrid approaches are feasible therapeutic approaches in high-risk patients and are associated with less severe complications and shorter hospitalization and recovery times. The long-term outcome (more than 10 years) compared to conventional surgery is still to be investigated.

## 4. Conclusions

An aortic pseudoaneurysm with secondary ABF should be suspected in all patients with previous history of aortic surgery addressing for hemoptysis or epistaxis. In our patient with associated SARS-CoV-2 pneumonia, increased intrathoracic pressure because of excessive cough could have contributed to the development of the ABF. Computed tomography is the most appropriate diagnostic tool and TEVAR should be considered the standard of care. When necessary, prior closed-chest debranching of aortic arch branches should be performed to ensure an adequate landing zone with good sealing of the lesion.

## Figures and Tables

**Table 1:** Summary of studies.

Study	Patients	Age, Sex	Symptoms	Age at Culprit Intervention	Type(s) of Surgery	Complication Responsible for ABF	Treatment	Outcome
Aslam et al., 2011 [21]	1	28, F	Hemoptysis	16	Resection of the native DTA, graft replacement of the DTA with proximal and distal end-to-side anastomoses	PSA proximal anastomosis	Replacement of DTA synthetic graft + wedge resection lung	38-years- old: new PSA at the caudal end of the graft treated with coil embolization and Amplatzer vascular plug, uneventful at 1 month
Bhuyan et al., 2007 [22]	1	45, M	Recurrent, massive haemoptysis	25	Synthetic patch aortoplasty	PSA aortoplasty site	Interposition of a synthetic graft	No follow-up data
Bozzani et al., 2017 [23]	1	65, M	Hemoptysis	48	Extra-anatomic bypass distal arch-DTA	PSA distal anastomosis	TEVAR between extra-anatomic bypass and distal DTA + Amplatzer occlusion of the proximal DTA at the level of the coarctation	Uneventful 15 months
Bugge et al., 2021 [24]	1	43, M	Dry cough, recurrent hemoptysis	32	1. 10-years-old: patch augmentation complicated with PSA 2. 15-years-old: new patch augmentation complicated with infection 3. 32-years-old: interposition of a synthetic graft	PSA distal anastomosis	TEVAR	New ABF after 2 months secondary to left main bronchus compression by the graft, treated with extra-anatomic bypass AA-DTA + left pulmonary hilum dissection with excision of the portion of the bronchus containing the fistula + lung autotransplantation, uneventful 6 months
Caes et al., 1993 [25]	1	24, M	Irritative cough, recurrent hemoptysis	11	Synthetic patch aortoplasty	PSA aortoplasty site	Interposition of a synthetic graft + closure of the pulmonary side of the ABF with running suture	Postoperative MSOF, discharged after 48 days, uneventful 1 year
Chuter et al., 1996 [26]	1	56, M	Massive hemoptysis	35	Interventions 1 and 2—unknown age and surgical technique, complicated with re-coarctation and PSA 3. 35-years-old: interposition of a synthetic graft	PSA distal anastomosis	TEVAR	Uneventful 7 months
Eldien et al., 2011 [12]	1	35, F	Hemoptysis	35	Interposition of a synthetic graft	Unspecified complication at distal anastomosis	Hybrid: TEVAR + left subclavian–to–left carotid bypass graft	Graft infection after 1 month treated with open surgical repair—extra-anatomic bypass AA—DTA + removal of the infected graft + lung decortication. Postoperative ECMO support for 13 days and MSOF, discharged after 62 days, uneventful 3 months
Eren et al., 2005 [18]	1	42, M	Recurrent hemoptysis	25	Synthetic patch aortoplasty	PSA aortoplasty site	Synthetic patch aortoplasty + debridement and suture of lung parenchima	No follow-up data
Fontana et al., 2018 [11]	1	46, M	Recurrent hemoptysis	18	Unspecificed surgical repair	PSA	TEVAR	Uneventful 30 days
Foster et al., 1998 [27]	1	62, M	Recurrent, massive hemoptysis	48	1. 18-years-old: resection of the coarctation and replacement with a homograft complicated with aneurysm formation 2. 44-years-old: interposition of a synthetic graft	PSA distal anastomosis	Interposition of a synthetic graft	Uneventful 4 years
Garniek et al., 1990 [28]	1	5, M	Recurrent hemoptysis	5 months	1. Unknown correction of PDA and coarctation in the neonatal period, complicated with occlusion of the subclavian graft at the age of 5 months 2. Extra-anatomic bypass AA-DTA	PSA distal anastomosis	-	Exitus—uncontrollable intrabronchial hemorrhage
Hamilton et al., 2004 [29]	3	28, F	Massive hemoptysis + dyspnea	7	Synthetic patch aortoplasty	PSA aortoplasty site	Interposition of a synthetic graft + fistula closure	No follow-up data
		37, F	Massive hemoptysis	20	Synthetic patch aortoplasty	PSA aortoplasty site	Interposition of a synthetic graft	No follow-up data
		57, F	Massive hemoptysis	24	Synthetic patch aortoplasty	PSA aortoplasty site	Interposition of a synthetic graft	No follow-up data
Hayat J, 2010 [30]	2	30, F	Massive hemoptysis	24	Synthetic patch aortoplasty	PSA aortoplasty site	Extra-anatomic bypass AA-DTA + ABF closure covered with pleura	Postoperative hemotorax, no follow-up
		46, M	Hemoptysis	38	Synthetic patch aortoplasty complicated with massive hemorrhage + paraplegia	PSA aortoplasty site	Interposition of a synthetic graft + ABF closure	No follow-up data
Kakos et al., 1975 [31]	1	56, M	Hemoptysis	49	Not mentioned	Not mentioned	Direct closure of the aorta	Uneventful 12 months
Kalkat et al., 2003 [32]	2	54, M	Recurrent hemoptysis + left sided chest pain	43	Synthetic patch aortoplasty complicated with massive hemorrhage + paraplegia	PSA aortoplasty site	Extra-anatomic bypass AA-DTA + resection of the involved aorta and PSA + bronchus repair	Uneventful 10 years
		52, M	Massive hemoptysis	22	2 interventions—synthetic patch aortoplasty at the age of 13 and 22 years complicated with paraparesis and transient cortical blindness	PSA aortoplasty site	Interposition of a synthetic graft	Uneventful 3 years
Kamler et al., 2001 [33]	1	38, M	Massive hemoptysis	28	1. 15-years-old: synthetic patch aortoplasty complicated with re-coarctation 2. 28-years-old: synthetic patch aortoplasty	PSA aortoplasty site	Interposition of a synthetic graft + left pneumonectomy	Uneventful 3 years
Kansal et al., 2015 [34]	1	46, M	Recurrent hemoptysis	17	Unknown open surgical repair	PSA	TEVAR	No follow-up data
Lawrence et al., 1997 [35]	1	23, F	Massive hemoptysis	15	Interventions 1 and 2—unknown age, surgical technique and complications. 3. 15-years-old: interposition of a synthetic graft	PSA	Extra-anatomic bypass AA-DTA+ removal of the old graft and PSA + bypass using a synthetic graft between the extra-anatomic conduit and the left axillary artery	No follow-up data
Manganas et al., 2001 [36]	1	29, F	Massive hemoptysis	24	1. 3-years-old: resection of the coarctation and end-to-end anastomosis complicated with re-coarctation 2. 9-years-old: synthetic patch aortoplasty complicated with recoarctation 3. 24-years-old: extra-anatomic bypass AA-DTA	PSA distal anastomosis	Unspecified surgical repair	No follow-up data
Marcheix et al., 2007 [37]	1	32, M	Massive hemoptysis	29	1. 4-years-old: synthetic patch aortoplasty, complication not mentioned 2. 29-years-old: interposition of a synthetic graft	PSA distal anastomosis	TEVAR	Uneventful 12.9 months
Milano et al., 1999 [38]	1	34, M	Recurrent hemoptysis + dysphonia	17	Synthetic patch aortoplasty	PSA aortoplasty site	Interposition of a synthetic graft + partial removal of the upper lobe of the left lung	Uneventful 8 months
Moore et al., 2018 [39]	1	67, F	Massive hemoptysis	Not mentioned	Unknown open surgical repair	PSA distal anastomosis	TEVAR	No follow-up data
Munneke et al., 2005 [14]	1	53, F	Recurrent hemoptysis	27	Synthetic patch aortoplasty	PSA aortoplasty site	TEVAR covering the origin of the left subclavian artery with retrograde flow through the left vertebral artery	No follow-up data
Neves et al., 2017 [40]	1	38, F	Massive hemoptysis + precordial pain	16	Extra-anatomic bypass AA-DTA	PSA distal anastomosis	TEVAR	Uneventful 3 months
Nodari et al., 2021 [13]	1	78, F	Recurrent hemoptysis	41	Extra-anatomic bypass left subclavian artery-DTA	PSA distal anastomosis	Hybrid approach: Exclusion of the extra-anatomic bypass with an Amplatzer plug + coil exclusion of the PSA + TEVAR of the native aorta + left axillo-femoral bypass to relieve pressure in the pre-coarctation aortic arch	Uneventful 4 months
O’Sullivan et al., 2014 [41]	1	46, M	Massive hemoptysis	13	1. Neonatal period: unknown correction 2. 13-years-old: extra-anatomic bypass distal arch-DTA with reimplantation of the left subclavian artery into the proximal end of the bypass graft	PSA left subclavian artery reimplantation site	Occlusion of the left subclavian artery with an Amplatzer plug + TEVAR exclusion of the extra-anatomic bypass	No follow-up data
Posacioglu et al., 2004 [4]	1	26, M	Massive hemoptysis	10	Synthetic patch aortoplasty	PSA aortoplasty site	Interposition of a synthetic graft + suture ligature of the pulmonary lesion	No follow-up data
Quintana et al., 2006 [42]	1	47, M	Massive hemoptysis	32	Unknown open surgical repair	PSA	TEVAR	Hemoptysis in the 5th postoperative day secondary to type 2B endoleak treated with coil embolization, uneventful at 12 months
Rodriguez-Caulo et al., 2011 [43]	1	39, M	Hemoptysis + atypical chest pain	12	Synthetic patch aortoplasty	PSA aortoplasty site	TEVAR	Proximal Type 1A endoleak the 3rd postoperative day: hybrid management with a secondary TEVAR + left carotid-left subclavian artery bypass, uneventful at 12 months
Saunders et al., 2002 [44]	1	37, M	Epistaxis + hemoptysis	19	Synthetic patch aortoplasty	PSA aortoplasty site	Interposition of a synthetic graft	Minor stroke in the postoperative period, no follow up
Sinelnikov et al., 2015 [45]	1	6, F	Hemoptysis + dyspnea	5	1. 5-years-old: interposition of a synthetic graft complicated with re-coarctation 2. 3 months after the 1rst intervention: balloon dilatation of the re-coarctation	PSA proximal anastomosis	Replacement of the interposed synthetic graft + repair of the left lower lobe bronchus	Uneventful 6 months
Smayra et al., 2001 [5]	1	61, M	Recurrent hemoptysis	43	Extra-anatomic bypass distal arch-DTA	PSA distal anastomosis	TEVAR between extra-anatomic bypass and distal DTA	Injury to the iliac artery while manipulating the catheter: iliofemoral bypass, uneventful 2 years
Verma et al., 2012 [46]	1	36, M	Recurrent hemoptysis + dysphonia	32	1. 23-years-old: synthetic patch aortoplasty complicated with ruptured PSA at aortoplasty site 2. 32-years-old: extra-anatomic bypass AA-DTA with ligation of the aortic arch distal to the left common carotid artery and of the distal descending thoracic aorta after the PSA complicated with persistent flow in the PSA and ABF	PSA aortoplasty site	Endovascular occlusion of the distal aortic arch with Amplatzer plug 2, of the aortic coarctation with Amender PDA closure device and of the distal leak site with Amender PDA closure device	Uneventful 6 months

AA—ascending aorta; DTA—descending thoracic aorta; PSA—pseudoaneurysm; MSOF—multiple systems organ failure; ECMO—extracorporeal membrane oxygenation.

**Table 2:** PSA with ABF: treatment and outcome.

Intervention (No. of Cases)	Complications (No. of Cases)
Replacement of the synthetic graft (1 case)	PSA (after 10 years) (1 case)
Interposition of a synthetic graft (12 cases)	MSOF (postoperative) (1 case) Minor stroke (postoperative) (1 case)
Synthetic patch aortoplasty (1 case)	-
Extra-anatomic bypass (3 cases)	Hemothorax (postoperative) (1 case)
Direct closure (1 case)	-
Unfinished intervention (1 case)	Exitus (intraoperative) (1 case)
Unspecified (1 case)	-
Endovascular (14 cases)	ABF (after 2 months) (1 case) Endoleak (Type 1A and 2B, postoperative) (2 cases) Iliac artery injury (intraoperative) (1 case)
Hybrid (2 cases)	Graft infection (after 1 month) (1 case)

## Data Availability

Data available on request.
